# Anaphylactic Shock as a Rare Side Effect of Intravenous Amiodarone

**DOI:** 10.7759/cureus.21118

**Published:** 2022-01-11

**Authors:** Aisha Batool, Khadija Batool, Hafsa Habib, Shahzad Chaudhry

**Affiliations:** 1 Internal Medicine, Columbia St. Mary’s Hospital, Milwaukee, USA; 2 Internal Medicine, Services Hospital Lahore, Lahore, PAK; 3 Internal Medicine, Nishtar Medical College, Multan, PAK; 4 Family Medicine, Aurora Health Center, Greenfield, USA

**Keywords:** side effects of amiodarone, allergic reaction to amiodarone, hypersensitivity, atrial fibrillation, anaphylactic reaction to intravenous amiodarone

## Abstract

Amiodarone is a very commonly used antiarrhythmic agent. However, it has a wide variety of systemic side effects as well as many hypersensitivity and allergic reactions, ranging from angioedema to anaphylactic shock in patients who have iodine allergies. We present a rare and unique case of an 86-year-old female who developed anaphylactic shock from intravenous (IV) amiodarone. She had no reported allergies to iodine or iodinated contrast. She had a history of chronic persistent atrial fibrillation and was being maintained on oral amiodarone as an outpatient. She was admitted with shortness of breath and was found to have atrial fibrillation with rapid ventricular response. She was started on an IV amiodarone bolus. Immediately after a few milliliters of infusion, she complained of shortness of breath, with facial flushing and generalized blanching erythema, followed by severe hypotension and cardiopulmonary arrest. IV amiodarone infusion was suspected to be the culprit and was discontinued immediately. IV epinephrine 0.3 mg was administered, followed by the advanced cardiovascular life support (ACLS) protocol for cardiopulmonary arrest. She did not respond to the standard ACLS protocol and continued to remain in cardiopulmonary arrest. A spot diagnosis of anaphylactic reaction to IV amiodarone was made, and she was started on IV epinephrine infusion 0.1 µg/kg/minute, and immediate return of spontaneous circulation was achieved. She was started on IV methylprednisolone 125 mg, IV famotidine 20 mg, and IV diphenhydramine 25 mg. She was intubated and required mechanical ventilation. She was successfully extubated later and safely discharged, receiving oral metoprolol 25 mg for rate control and PO rivaroxaban 20 mg once daily.

Anaphylactic shock from IV amiodarone administration is a potentially fatal complication observed in patients with prior reported allergies to iodine or iodinated contrast media. It has rarely been reported in the absence of prior allergy to iodine or iodinated contrast media. Prompt recognition by clinicians is prudent for early diagnosis and appropriate treatment.

## Introduction

Amiodarone is a very effective class IIIa antiarrhythmic agent (potassium channel modulator) [1], with a wide variety of side effects. It comes with a black box warning of hepatic impairment, pulmonary toxicity, and worsened arrhythmia [2-5]. Hypersensitivity reactions to amiodarone ranging from angioedema to anaphylactic shock have been reported in patients with iodine allergies [6-10]. Anaphylactic shock from intravenous (IV) amiodarone administration is a potentially fatal complication in patients with prior reported allergies to iodine or iodinated contrast media [11,12]. However, it has been rarely reported in the absence of prior allergy to iodine or iodinated contrast media, and in such circumstances, the allergic reaction is attributed to the polysorbate-80 content of the IV formulation [13,14]. Prompt recognition by clinicians is prudent for early diagnosis and appropriate treatment. Alternative strategies should be utilized for the treatment of atrial fibrillation in such patients [15,16].

## Case presentation

**Figure 1:**
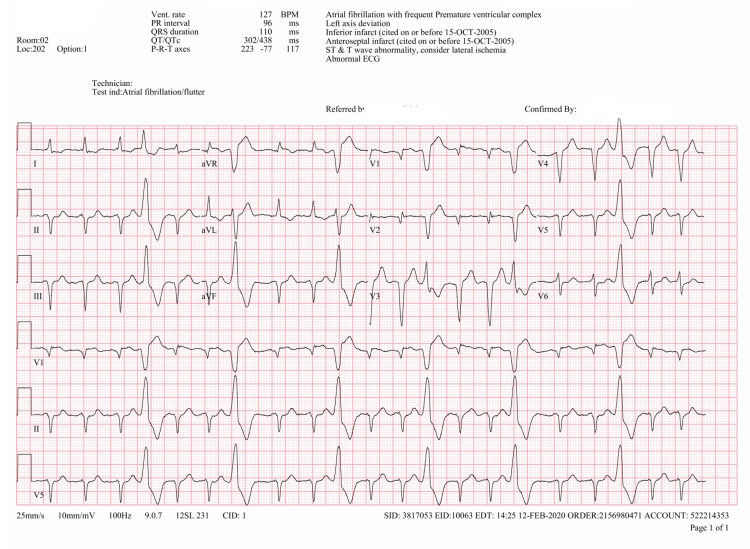
Admission electrocardiogram consistent with atrial fibrillation with rapid ventricular rate

The patient was an 86-year-old Caucasian female with a history of chronic atrial fibrillation maintained as an outpatient on PO amiodarone 200 mg once daily for rate control and PO rivaroxaban 20 mg once daily. She had no allergies to iodine or iodinated contrast media, and she had been exposed to iodinated contrast media in the recent past without any allergic reaction. The patient presented to the emergency room with shortness of breath and flu-like symptoms. She reported subjective fever and chills. In the emergency room, her vital signs were as follows: temperature, 39°C; heart rate, 128 beats/minute; respiratory rate, 22 breaths/minute; blood pressure, 126/86; and oxygen saturation, 96% on room air. She underwent a detailed workup, including an influenza swab, which was negative. However, a viral respiratory nucleic acid amplification test was positive for human metapneumovirus. This established the diagnosis of viral upper respiratory tract infection. She was admitted to the hospital with a diagnosis of viral upper respiratory tract infection causing atrial fibrillation with rapid ventricular response. The 12-lead electrocardiogram (EKG) image is shown in Figure 1.

The patient was seen by cardiac electrophysiology and started on an IV amiodarone bolus 150 mg. Within a few milliliters of the initiation of the bolus, she complained of severe shortness of breath and chest pain. She became unresponsive, was initially found hypotensive, and soon became pulseless. IV amiodarone was stopped immediately, and she was started on the advanced cardiovascular life support (ACLS) protocol, including cardiopulmonary resuscitation and endotracheal intubation. She was given IV epinephrine 0.3 mg as well as standard ACLS care, but she remained pulseless. She was noted to have flushing of the face and generalized blanching erythema of the whole body. A spot diagnosis of amiodarone infusion-related anaphylactic shock was made, and she was started on IV epinephrine infusion 0.1 µg/kg/minute.

**Figure 2:**
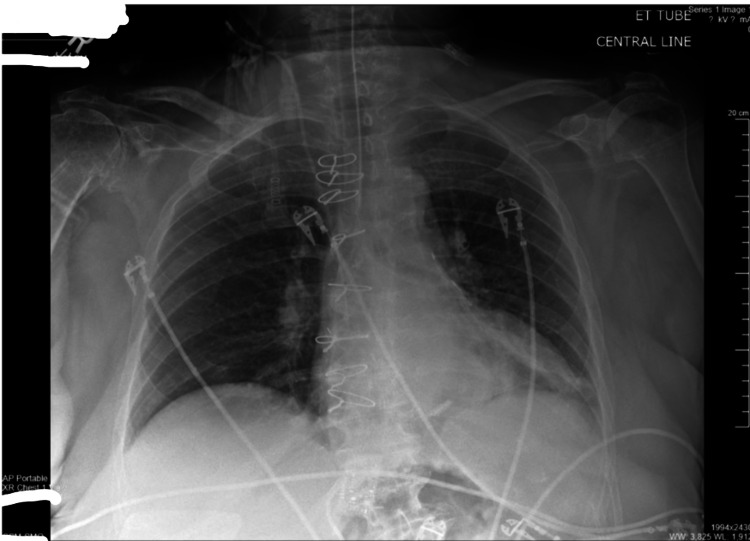
Chest X-ray

**Figure 3:**
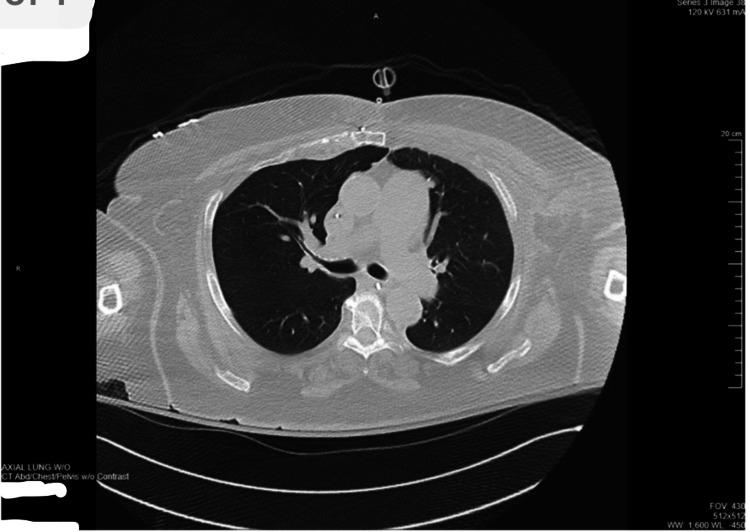
CT scan of the chest with IV contrast

Return of spontaneous circulation was achieved immediately. She was started on supportive care for anaphylactic reaction, including IV methylprednisolone 125 mg, immediately followed by IV H1 + H2 blockers (diphenhydramine 25 mg and famotidine 20 mg). During the cardiopulmonary arrest, she was intubated and maintained on mechanical ventilation. Chest X-ray and computed tomography revealed multiple bilateral rib fractures and chest wall hematomas (Figure 2 and Figure 3).

Two days later, she was successfully extubated. Her hemoglobin level decreased, requiring suspension of rivaroxaban treatment and transfusion of one unit of packed red blood cells. She was transitioned to IV heparin infusion in lieu of anemia and was successfully switched to oral rivaroxaban 20 mg once daily prior to discharge. Steroids were slowly tapered off as well. She was safely discharged home thereafter. On her post-discharge follow-up, she was started on oral amiodarone 100 mg daily with good tolerance.

## Discussion

Atrial fibrillation is the most commonly diagnosed arrhythmia. As the world population ages, cases of atrial fibrillation continue to rise. Atrial fibrillation is predicted to affect 17.9 million people in Europe by 2060 and 6-12 million people in the United States by 2050 [17,18]. Multiple pharmacologic and nonpharmacologic strategies have been utilized to achieve rate and rhythm control in patients with atrial fibrillation. Amiodarone is a class IIIa antiarrhythmic medication (potassium channel modulator) utilized in both oral and IV formulations. Its mechanism of action involves prolonging repolarization by blocking sodium channels and calcium channels in cardiac myocytes. It causes delayed phase 3 repolarization and effective refractoriness by blocking potassium channels [1]. It also inhibits adrenergic stimulation and decreases conduction through the atrioventricular node [2-5]. It comes with a black box warning of pulmonary toxicity, liver toxicity, and proarrhythmic effects [11]. It is one of the most effective pharmacologic means of both rate and rhythm control. It has a wide variety of side effects, involving almost every organ in the body. Allergic reactions to amiodarone have been reported, which vary in severity and type, ranging from hypersensitivity to angioedema and anaphylactic shock. Most allergic reactions occur in patients with allergies to iodine or iodinated contrast media. Both oral and IV formulations of the drug contain iodine; the iodine content is 75 mg in a 200 mg tablet of amiodarone and 18.7 mg/mL in IV solution. Approximately 10% of the iodine content of oral amiodarone is released into the circulatory system and may increase the risk of hypersensitivity reactions in iodine-sensitive patients [12]. However, there are anecdotal reports evaluating the use of amiodarone in patients with allergies to iodine or iodinated contrast media. The incidence of hypersensitivity in such cases was reported to be <1%. Therefore, documented allergy to iodine is not a contraindication to amiodarone administration, and most patients with such allergies are able to tolerate amiodarone [19]. Anaphylactic shock from IV amiodarone administration is a potentially fatal complication observed in patients who have had prior reported allergies to iodine or iodinated contrast media [11,12]. However, anaphylactic shock has rarely been reported in the absence of prior allergies to iodine or iodinated contrast media, and in such circumstances, the allergic reaction is attributed to the polysorbate-80 (also known as polyoxyethylene-sorbitan-20-monooleate) content of the IV formulation [13,14]. Polysorbate-80 is a solubilizing agent ubiquitously used in medical preparations, including certain vaccines, such as the human papillomavirus vaccine, as well as IV formulations of amiodarone. Some cases of hypersensitivity reactions to this agent have been reported [13,14]. However, our patient did not have a reported allergy to polysorbate-80 and had never received IV amiodarone in the past. We did not assume that she has an allergy to polysorbate-80 as we have not done any formal testing for this. This case is unique, as she was started back on PO amiodarone with documented good tolerance.

## Conclusions

Amiodarone is a very effective antiarrhythmic agent; however, it has a wide variety of systemic side effects, and their treatment is well established in the literature. This case is a reminder to look for life-threatening allergic reactions to IV amiodarone in patients who have been taking oral amiodarone. Prompt recognition by clinicians is prudent for early diagnosis and appropriate treatment to save a patient’s life. Alternative strategies should be utilized for the treatment of atrial fibrillation in such patients. It is, however, recommended to give a trial of oral amiodarone if both the clinician and patient are in agreement. However, intravenous amiodarone must be avoided in these patients.

## References

[1] Lei M, Wu L, Terrar DA, Huang CL (2018). Modernized classification of cardiac antiarrhythmic drugs. Circulation.

[2] (2019). Heart Rhythm Society: 2019 AHA/ACC/HRS focused update of the 2014 AHA/ACC/HRS guideline for the management of patients with atrial fibrillation. https://www.hrsonline.org/guidance/clinical-resources/2019-ahaacchrs-focused-update-2014-ahaacchrs-guideline-management-patients-atrial-fibrillation.

[3] Miller JM, Zipes DP (2005). Therapy for cardiac arrhythmias. Braunwald's heart disease: a textbook of cardiovascular medicine, 7th edition.

[4] van Erven L, Schalij MJ (2010). Amiodarone: an effective antiarrhythmic drug with unusual side effects. Heart.

[5] Jafari-Fesharaki M, Scheinman MM (1998). Adverse effects of amiodarone. Pacing Clin Electrophysiology.

[6] Lahiri K, Malakar S, Sarma N (2005). Amiodarone-induced angioedema: report of two cases. Indian J Dermatol Venereol Leprol.

[7] Burches E, Garcia-Verdegay F, Ferrer M, Pelaez A (2000). Amiodarone-induced angioedema. Allergy.

[8] Kurt İH, Yalcin F (2012). Anaphylactic shock due to intravenous amiodarone. Am J Emerg Med.

[9] Fransi S, Briedis J (2004). Anaphylaxis to intravenous amiodarone. Anaesth Intensive Care.

[10] Stafford L (2007). Hypersensitivity reaction to amiodarone in a patient with a previous reaction to an iodinated radiocontrast agent. Ann Pharmacother.

[11] (2003). Cordarone X 150 mg/3 ml solution for injection. Macquarie Park, NSW: Sanofi-Aventis.

[12] Brouse SD, Phillips SM (2005). Amiodarone use in patients with documented allergy to iodine-containing compounds. Pharmacotherapy.

[13] Coors EA, Seybold H, Merk HF, Mahler V (2005). Polysorbate 80 in medical products and nonimmunologic anaphylactoid reactions. Ann Allergy Asthma Immunol.

[14] Badiu I, Geuna M, Heffler E, Rolla G (2012). Hypersensitivity reaction to human papillomavirus vaccine due to polysorbate 80. BMJ Case Rep.

[15] Stiell IG, de Wit K, Scheuermeyer FX (2021). 2021 CAEP acute atrial fibrillation/flutter best practices checklist. CJEM.

[16] January CT, Wann LS, Alpert JS (2014). 2014 AHA/ACC/HRS guideline for the management of patients with atrial fibrillation: a report of the American College of Cardiology/American Heart Association Task Force on Practice Guidelines and the Heart Rhythm Society. Circulation.

[17] Krijthe BP, Kunst A, Benjamin EJ (2013). Projections on the number of individuals with atrial fibrillation in the European Union, from 2000 to 2060. Eur Heart J.

[18] Chugh SS, Havmoeller R, Narayanan K (2014). Worldwide epidemiology of atrial fibrillation: a Global Burden of Disease 2010 Study. Circulation.

[19] Lakshmanadoss U, Lindsley J, Glick D, Twilley CH, Lewin JJ, Marine JE (2011). Incidence of amiodarone hypersensitivity reaction in hospitalized patients with prior allergic reaction to iodine or iodinated contrast. Circulation.

